# Mitochondrial genome of *Sitona callosus* (Coleoptera: Curculionidae) and phylogenetic analysis within Entiminae

**DOI:** 10.1080/23802359.2017.1365657

**Published:** 2017-08-17

**Authors:** Li Zhang, Juan Wang, Xing-Zhuo Yang, Xiao-Peng Li, Run-Qiu Feng, Ming-Long Yuan

**Affiliations:** State Key Laboratory of Grassland Agro-Ecosystems, College of Pastoral Agricultural Science and Technology, Lanzhou University, Lanzhou, People’s Republic of China

**Keywords:** Beetles, weevils, *Sitona callosus*, mitochondrial DNA, phylogeny

## Abstract

In this study, we sequenced and annotated the nearly complete mitochondrial genome (mitogenome) of *Sitona callosus* (Coleoptera: Curculionidae). This mitogenome was 14,333 bp long and encoded 13 protein-coding genes, 19 transfer RNA genes (tRNAs), and two ribosomal RNA unit genes. Gene rearrangements were presented in a tRNA cluster of six tRNAs between *nad3* and *nad5*, i.e. the ancestral order ARNSEF was changed to be RNSAEF. All tRNAs had a typical secondary cloverleaf structure, except for *trnS1* which lacked the dihydrouridine arm. The Bayesian phylogenetic tree of 11 Entiminae species based on the concatenated nucleotide sequences of 13 PCGs showed that *S. callosus* and *S. lineatus* formed a clade which was at the basal position in the Entiminae phylogeny.

## Introduction

*Sitona callosus* is an important insect pest on many leguminous plants in China. Here, we sequenced and annotated the mitochondrial genome (mitogenome) of *S. callosus*, following the methods of Yuan et al. (2016). Adult specimens were collected from Huining County, Gansu Province, China, in July 2014. Samples have been deposited in College of Pastoral Agricultural Science and Technology, Lanzhou University, Lanzhou, China. The *S. callosus* mitogenome was amplified with a set of universal and specific primer pairs (available from corresponding author on request) and sequenced in both directions.

We obtained the nearly complete mitogenome of *S. callosus*, with 14,333 bp long (GenBank accession number MF594624). The region that we failed to sequence in *S. callosus* was located between *rrnS* and *nad2*. This area in insect mitogenomes generally contains notable base composition bias, high numbers of tandem repeats, and stable stem-loop structures, which could result in disruption of PCR and sequencing reactions, as reported in other coleopterans (Haran et al. 2013). This mitogenome encoded 13 protein-coding genes (PCGs), 19 transfer RNA genes (tRNAs), the large and small ribosomal RNA unit genes (*rrnL* and *rrnS*). The order and orientation of the mitochondrial genes are identical to the inferred ancestral arrangement of insects (Boore 1999), except for a tRNA rearrangement in a cluster of six tRNAs between *nad3* and *nad5*. Typically, the ancestral order of the six tRNAs is ARNSEF, whereas *S. callosus* exhibited RNSAEF, as reported in *Sitona lineatus* (Haran et al. 2013). Two large gene overlaps, i.e. *atp8*/*atp6* (−7 bp) and *nad4*/*nad4L* (−7 bp), were present in the *S. callosus* mitogenome, whereas a total of 72 bp intergenic spacers were found in 12 positions, ranging in size from 1 to 29 bp. The *S. callosus* mitogenome with an A + T content of 76.37% presented a positive AT-skew (0.049) and a negative GC-skew (−0.169) on the J-strand. Among the 13 PCGs, the lowest A + T content was 68.56% in *cox1*, while the highest was 85.62% in *atp8*. Ten PCGs started with a typical ATN codon: one (*nad6*) with ATC, two (*nad2* and *nad3*) with ATA, two (*cox2* and *atp8*) with ATT, five (*atp6*, *cox3*, *nad4*, *nad4L*, and *cob*) with ATG. The remaining three PCGs started with TTG (*nad1*), GTG (*nad5*), or AAT (*cox1*). Four PCGs terminated with TAA or TAG, whereas the remaining nine terminated with an incomplete stop codon TA or T. All of the 19 tRNAs, ranging from 63 bp (*trnC*) to 71 bp (*trnK*), had a typical cloverleaf secondary structure, except for *trnS1* (AGN) in which its dihydrouridine arm simply formed a loop.

**Figure 1.**
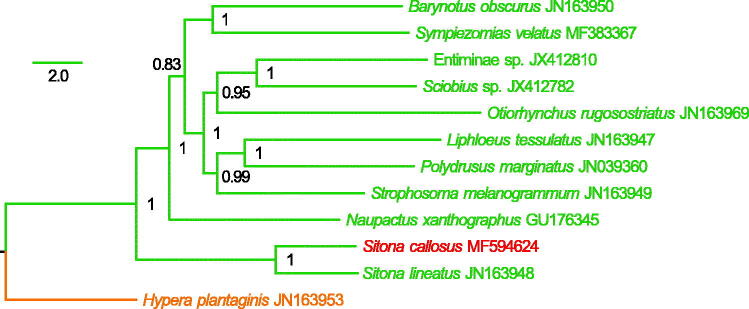
Mitochondrial phylogeny of 11 Entiminae species based on the concatenated nucleotide sequences of 13 mitochondrial protein-coding genes.

We conducted a Bayesian phylogenetic analysis in MrBayes 3.2.6 (Ronquist et al. 2012), using the concatenated nucleotide sequences of 13 PCGs from 11 Entiminae species and an outgroup from the subfamily Hyperinae (*Hypera plantaginis*). We determined the optimal partitioning schemes and corresponding nucleotide substitution models by PartitionFinder v1.1.1 (Lanfear et al. 2012). As shown in Figure 1, *S. callosus* clustered with *S. lineatus* with a high support value (posterior probability =1) and the *Sitona* clade was at the basal position in the Entiminae phylogeny.
